# The Forgotten History of Bacteriophages in Bulgaria: An Overview and Molecular Perspective on Their Role in Addressing Antibiotic Resistance and Therapy

**DOI:** 10.3390/v18010038

**Published:** 2025-12-25

**Authors:** Nikolay Kalvatchev, Tannaz Khanbabapour, Arit Sakkeer, Iliya Tsekov, Yancho Delchev, Tanya Strateva

**Affiliations:** 1 Department of Medical Microbiology, Faculty of Medicine, Medical University of Sofia, 1431 Sofia, Bulgaria; nkalvat@medfac.mu-sofia.bg (N.K.); dr.strateva@abv.bg (T.S.); 2 Faculty of Medicine (Foreign Students), Medical University of Sofia, 1431 Sofia, Bulgaria; 118426@students.mu-sofia.bg; 3 Virology Laboratory, Nadezhda Women’s Health Hospital, 1330 Sofia, Bulgaria; 4 Clinic of General and Oncological Gynecology, Military Medical Academy, 1606 Sofia, Bulgaria; yanchodelchev@abv.bg

**Keywords:** Bacteriophages, Bulgaria, antibiotic resistance, lytic activity, cytotoxicity

## Abstract

Bacteriophages, often referred to as “bacteria eaters,” have gained renewed interest as a powerful alternative to traditional antibiotics, particularly in addressing antibiotic-resistant bacterial infections. The present review summarizes data collected in Bulgaria during the 1960s, 1970s, and 1980s, drawing connections between past findings and present-day understanding of cytotoxicity and the clinical validation of bacteriophage applications. Its sections describe phage structure, mechanisms of action, and historical findings both globally and within the Bulgarian context, while also highlighting emerging trends and applications. The cited studies delve into the past through contemporary research contributions related to “Bulgarian phages”, a topic that remains underexplored in existing literature. The role of phages in medical microbiology is discussed alongside the challenges of therapeutic implementation, with particular focus on insights gained from the Bulgarian experience. In conclusion, by fostering international collaborations, investing in infrastructure, and establishing supportive policies, bacteriophage therapy can emerge as a critical tool for managing bacterial infections and reducing the global burden of antibiotic resistance in the future.

## 1. Introduction

Bacteriophages (from Greek: “devourers”), being the most abundant biological entities on Earth, are viruses that infect and replicate within bacteria with high specificity. These naturally existing life forms have garnered significant attention in microbiology and toxicology due to their potential applications in bacterial control and their profound impact on bacterial dynamics and host interactions [1,2]. Bacteriophages (phages) are ubiquitous in the environment, found in soil, rivers, lakes, oceans, and even in the gastrointestinal tracts of living organisms [3]. Exhibiting targeted activity in contrast to the broad spectrum of antibiotics while sparing beneficial microbiota, phages have sparked renewed interest in their therapeutic potential, especially with the rise of antibiotic resistance among bacterial pathogens worldwide [4,5].

The history of bacteriophage therapy dates back from 1919 in France when French-Canadian microbiologist Félix d’Hérelle first managed to cure several children with severe dysentery using bacteriophages isolated from stool samples. This interest in phage therapy later expanded considerably throughout 1920 and into the 1930s, which lead to the development and commercialization of various phage preparations [6]. However, following the discovery of antibiotics, interest in bacteriophages faded. The discovery of new groups of antimicrobial agents in the 1950s and 1960s and their improvements in the 1970s and 1980s somewhat diminished concerns over antibiotic resistance. Since the 1990s, however, the synthesis of new antimicrobial agents has slowed significantly, while antibiotic resistance has risen. In 2001, the World Health Organization (WHO) formally raised the issue of antibiotic resistance and introduced a global strategy for combating antimicrobial resistance. By 2014, WHO warned that the world was heading into a so-called “post-antibiotic era”. Consequently, the demand for effective alternative antibacterial treatments has surged, with bacteriophages emerging as a promising solution in this context [7,8,9].

While many countries, particularly in the West, are advancing in the regulation and clinical implementation of phage therapy [10], Bulgaria’s contribution to this field remains scarce. Although bacteriophage research has been relatively limited in Bulgaria, historical works conducted as far back as the 1960s provide valuable insights. The present review article marks the first time these reports being discussed and referenced online, shedding light on research that was previously only available within specific institutional archives at the Medical University of Sofia, Bulgaria. These studies are included in the following works: “Production of Anthrax Bacteriophages and Development of Their Application in Diagnostics” [11], “Isolation and Study of *Klebsiella pneumoniae* Bacteriophages with Regard to Their Application in Epidemiology Practice” [12], “Phagotyping of *S. enteritidis* Using a Set of Bacteriophages”, and “Isolation, Study, and Application in *Enterococci* Diagnostics” [13]. These early investigations laid the groundwork for bacteriophage isolation and their practical application, addressing significant gaps in our understanding of their role in epidemiology and diagnostics. Building on this foundation, recent but limited research efforts in Bulgaria have continued to evaluate the safety and effectiveness of phages in combating major bacterial pathogens, aspects of which are also evaluated to some extent in this review.

From a therapeutic perspective, bacteriophages hold significant promise due to their immediate and direct bactericidal action. They are particularly suited for treating localized infections, such as chronic wounds or diabetic ulcers [14]. However, the role of temperate phages in enhancing the pathogenic potential of bacteria highlights the need for a rational approach to phage therapy. This includes mandatory sensitivity assessments of infectious agents to bacteriophages prior to use and the utilization of only highly virulent (lytic) phages for therapy [15]. Such an approach ensures the targeted and safe application of phages, maximizing their efficacy while minimizing potential risks. Furthermore, the cytotoxicity of bacteriophage preparations on human cells is a critical consideration. Studies focusing on the interaction between bacteriophages and mesenchymal stem cells have confirmed their safety, even when used at different concentrations, an important factor for their clinical viability [16]. These insights highlight the necessity of customizing phage therapy to match specific bacterial strains for the best therapeutic results. As more phage-based products become commercially available, careful evaluation of their lytic effectiveness and cytotoxic potential on an individual basis remains vital. Personalized approaches are essential to fully harness the capabilities of phage therapy, providing a safe and targeted alternative in the fight against antibiotic-resistant bacterial infections.

### 1.1. Bacteriophage Structure and Its Role in Phage Therapy

#### 1.1.1. Overview of Bacteriophage Structure

In terms of therapeutic, lytic phages are particularly valuable due to their ability to eradicate bacterial hosts through cell lysis. This sets them apart from lysogenic phages, which integrate their genetic material into bacterial genomes and, in some cases, facilitate the horizontal transfer of antibiotic resistance or virulence genes. Many Bacteriophages, in particular tailed phages, consist of a capsid (head) that encloses their genetic material (DNA or RNA) and a tail structure that mediates infection by delivering the genome into the bacterial host [17]. The tail fibers are crucial for host specificity, binding to bacterial surface receptors to initiate infection. Recent studies on *Acinetobacter* bacteriophage tail fibers have highlighted not only their essential role in targeting resistant bacterial strains but also their involvement in capsule degradation, receptor recognition, and enhancement of phage infectivity, reinforcing their therapeutic potential [18,19]. Their high host specificity allows lytic phages to selectively infect bacterial pathogens, including some antibiotic-resistant ones, while mostly sparing the commensal microbiota [20]. Additionally, their exclusively lytic nature prevents horizontal gene transfer, minimizing the risks commonly associated with phage therapy. Moreover, not all lytic phages are structurally identical; variations in tail fiber architecture and overall morphology can significantly influence how phages recognize hosts and survive under different environmental conditions [21]. Table 1 illustrates key structural variations among some of the common lytic phage families and highlights how these features impact their biological function.

**Figure 1:**
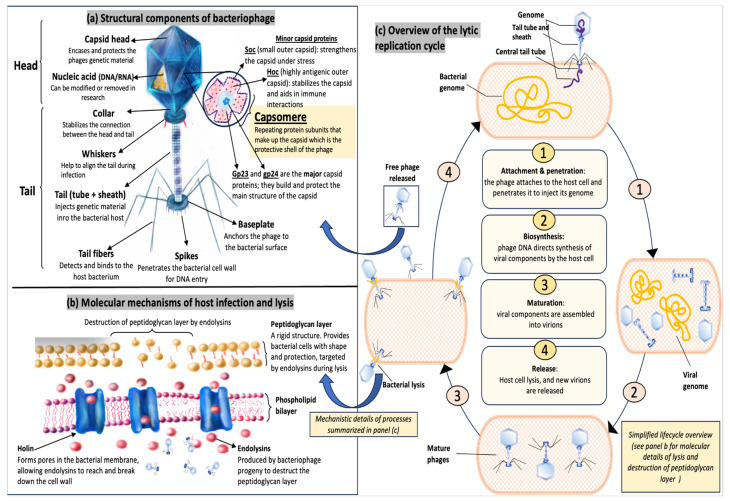
Schematic representation of the bacteriophage structure using phage T4 as a model. (**a**) Bacteriophage Structure: The bacteriophage consists of a capsid (head) and a tail structure essential for infection. The capsid, primarily made of gp23, encloses the viral genome, with gp24 reinforcing its stability. Hoc and Soc proteins further stabilize the capsid, with Hoc potentially facilitating interactions with host cells and Soc providing structural resilience. The tail structure, composed of a contractile sheath, a central tube, and tail fibers, is crucial for host recognition and DNA delivery. (**b**) Infection Mechanism: The phage attaches to bacterial receptors via long and short tail fibers, ensuring specificity. Upon binding, the tail sheath contracts, allowing the tail tube to pierce the bacterial membrane and inject phage DNA into the host cytoplasm. (**c**) Lytic Cycle: Inside the host, phage DNA directs the synthesis of viral components. As virions mature, phage-encoded enzymes degrade the bacterial cell wall, leading to lysis and the release of newly formed phages, which continue the cycle by infecting new bacterial hosts.

The capsid structure of the model phage T4 (Figure 1a) plays an integral role in maintaining phage stability and infectivity. The major capsid protein gp23 of T4 provides the primary structural framework, while gp24 reinforces the vertices to ensure capsid integrity [17]. The accessory proteins Hoc and Soc contribute to structural stability, with Hoc potentially influencing phage-host interactions and Soc acting as a stabilizer under stress conditions [22]. Although these proteins are not directly involved in bacterial lysis, their role in ensuring capsid robustness is crucial for phage survival and therapeutic application. A recent study by Majewska et al. (2023) [23] assessed the significance of gp24, Hoc, and Soc in phage stability under varying pH, temperature, and enzymatic exposure. Their findings demonstrated that the absence of gp24 and Soc weakened capsid integrity, ultimately reducing lytic efficiency. These findings emphasize the importance of capsid proteins in ensuring phage viability, particularly in therapeutic applications where environmental stability is crucial. Additionally, Miernikiewicz et al. (2012) [24] investigated the structural properties of T4 phage capsid proteins and found that proper capsid assembly and folding are essential for effective phage maturation and infectivity. While this study did not directly assess antibacterial resistance, its findings reinforce the broader importance of structural proteins in phage stability.

**Table 1:** Structural diversity among major morphological types of phages and their functional implications. Summary of the key morphological differences between some bacteriophage groups highlighting variations in tail structure, particle architecture, and their impact on phage function. Tail morphology influences DNA injection mechanisms, host range specificity, and performance in different therapeutic or environmental conditions.

Morphological Type	Tail Structure	Key Morphological Features	Functional Implications
Myovirus	Long, contractile tail	Large head, thick contractile tail [23]	Strong infection force; can inject DNA thought thick capsules; often broad host range [24,25]
Siphovirus	Long, non-contractile tail	Flexible, thin tail; small to medium head [23]	Slower DNA injection, typically narrower host range; often more stable in certain conditions [25]
Podovirus	Short, stubby tail	Very small tail; compact structure [23]	Fast DNA injection; often used in synthetic phage platforms; efficient in dense environments [24]
Inovirus	Filamentous (no head-tail)	Long, thread-like; non-lytic release [23]	Typically temperate; release without lysis; not used therapeutically due to integration risk [25]

Collectively, these insights highlight the crucial role of capsid integrity in phage infectivity and therapeutic effectiveness. Interaction between capsid stability and tail-mediated infection is fundamental to phage therapy against antibiotic-resistant bacteria. Understanding these structural elements can aid in optimizing engineered phages for clinical applications.

#### 1.1.2. Mechanisms of Action

Bacteriophage therapy continues to be rigorously evaluated for its efficacy and safety, particularly in addressing antibiotic-resistant bacterial infections and its potential applications in wound healing [25]. The mechanism of action underlying phage therapy begins with the recognition and attachment of phage tail fibers to specific bacterial surface receptors, as shown in Figure 1b. Recent high-resolution structural analyses have provided atomic-level insights into this process; for example, studies have revealed the conformational changes in phage lambda tail fibers upon binding to its *E. coli* receptor, a critical step that triggers DNA injection into the host cell [26,27]. These analyses have shown that receptor binding induces a substantial reorientation of the tail fiber domains, transitioning the fiber from a flexible, scanning state into a locked conformation necessary for stable attachment. This structural rearrangement not only ensures firm anchoring to the bacterial surface but also initiates allosteric signaling that propagates through the phage baseplate, leading to sheath contraction and DNA release [26]. Moreover, conserved motifs within the tail tip proteins suggest an evolutionary preservation of receptor-binding strategies among lambda-like phages [27]. Following stable attachment and conformational triggering, the phage injects its genetic material into the host cell (Figure 1c), a process often facilitated by virion-associated lysins (VALs) that locally degrade regions of the bacterial cell wall to ease genome delivery [28]. Once inside, the phage hijacks the host’s machinery to produce viral components. During the later stages of infection, specialized lytic enzymes known as endolysins play a critical role. Produced within the bacterial cytoplasm during replication, these enzymes, with assistance from holins that create pores in the cytoplasmic membrane—access the peptidoglycan layer [29]. Endolysins exhibit diverse enzymatic activities: amidases cleave bonds between the peptide stems and glycan backbones; muramidases (lysozyme-like enzymes) target the NAM-NAG glycan linkages; endopeptidases disrupt peptide cross-links; and glucosaminidases degrade NAG residues in the backbone [29,30]. The coordinated action of these enzymes compromises the integrity of the bacterial cell wall, causing osmotic imbalance and eventual lysis. In Gram-negative bacteria, this cascade occurs within the periplasmic space, ultimately leading to the release of newly formed phage progeny into the surrounding environment [28].

Furthermore, the rise of antibiotic resistance in bacteria is largely fueled by spontaneous mutations, horizontal gene transfer of resistance genes, and the enhancement of defense mechanisms like efflux pumps and biofilm production-factors that collectively reduce the effectiveness of standard antibiotic treatments [31,32]). Studies have linked these mechanisms of action to combatting antibiotic-resistant bacteria. For example, the enzyme OBPgp279, derived from the *Pseudomonas fluorescens* phage OBP, hydrolyzes peptidoglycan and penetrates the outer membranes of Gram-negative bacteria without requiring auxiliary proteins [33]. Engineered artilysins, which combine endolysins with membrane-penetrating peptides, have shown promising efficacy against multidrug-resistant (MDR) Gram-negative pathogens by overcoming the protective outer membrane barrier that typically limits endolysin activity [34,35]. Recent developments have introduced engineered lysins such as LysJEP8, which demonstrated strong antimicrobial activity against key Gram-negative ESKAPE pathogens, notably *Pseudomonas aeruginosa*, *Acinetobacter baumannii*, and *Klebsiella pneumoniae*, and effectively disrupted *P. aeruginosa* biofilms [36]. Similarly, artilysins like Art-175 have been engineered by fusing endolysins with polycationic peptides, achieving significant bacterial reduction against MDR strains [33]. In addition, CF-370, a next-generation engineered lysin, has shown broad-spectrum activity against clinically relevant Gram-negative bacteria, further highlighting the potential of structural modifications to enhance therapeutic efficacy [37]. Furthermore, recent bioengineering strategies, such as peptide fusion, domain shuffling, and rational design, have been emphasized for broadening the activity of endolysins against both Gram-negative and Gram-positive targets [38].

Complementing these advances, phages from the *Autographiviridae* family have demonstrated potential in regulating infections caused by *K. pneumoniae* and *A. baumannii*, with synergistic effects observed when used alongside conventional antibiotics [39]. Engineered phage-derived lytic enzymes (PLEs) have also been tailored for enhanced stability and broader antibacterial spectra through techniques such as domain fusion and chimeric construction. For instance, hybrid enzymes like ClyR have exhibited broad lytic activity, while modified virion-associated lysins (VALs) have shown improved specificity, particularly against Gram-positive bacteria [28,40]. Collectively, these advancements highlight the therapeutic potential of bacteriophages and their derivatives as cytotoxicity-safe, highly targeted tools for combating antibiotic-resistant bacterial infections.

### 1.2. Historical Background

#### 1.2.1. Discovery and Origin of Bacteriophages

In the summer of 1915, during World War I, a group of soldiers stationed on the outskirts of Paris suffered from an outbreak of severe hemorrhagic dysentery. The investigation of this outbreak was assigned to a young French-Canadian scientist, Félix d’Hérelle. Working in Paris at the prestigious Pasteur Institute, he quickly isolated and identified *Shigella* as the cause of the soldiers’ dysentery. While working with fecal samples from the patients, he observed that bacterial cultures would not grow, but rather lyse. Drops from already lysed cultures destroyed neighboring bacterial colonies. After filtration through an antibacterial filter, the mysterious antibacterial effect persisted. In 1917, d’Hérelle presented his findings to the French Academy of Sciences, hypothesizing that the lysis was caused by an “invisible microbe,” an antagonist to the dysentery bacillus. He introduced the term “bacteriophage” to describe the presumed bacterial lysis (from Greek “phagein,” meaning “to eat”) [41,42].

Not long after d’Hérelle’s work was published, other researchers asserted that they had observed comparable phenomena earlier. In 1915, British military bacteriologist Frederick Twort described an infectious agent capable of passing through filters and spreading between bacterial colonies. Meanwhile, Jules Bordet and André Gratia from the Pasteur Institute in Brussels proposed that bacteriophages were self-replicating bacterial enzymes, challenging d’Hérelle’s view that they were viruses. This scientific debate persisted well into the 1920s [41,42].

Bacteriophages also attracted significant interest among Russian scientists. Russian-Ukrainian microbiologist Nikolai Gamaleya first observed the phenomenon of lysis in plague bacteria in 1898. Following the Russian translation of d’Hérelle’s monograph *Bacteriophage*: Its Role in Immunity in 1926, the pioneer of cellular immunity, Ilya Mechnikov, was impressed. He spent years in France, establishing direct links between Russian microbiologists and French institutions. Georgian scientist George Eliava, who worked with d’Hérelle in Paris between 1918 and 1920, founded the first bacteriological laboratory dedicated to bacteriophages in Tbilisi. As a supporter of d’Hérelle, Eliava was among the first proponents of the viral theory of bacteriophages. In subsequent years, many other centers in Moscow, Leningrad, and Kharkiv engaged in phage research, and Lev Tarasevich, director of the Institute for Vaccines and Sera Control began extensive collaboration with French scientists in this field [41,42].

In his early works, d’Hérelle emphasized the relationship between bacteriophages, bacteria, and the infected organism, developing a theory of bacteriophages as factors of anti-infectious immunity. He proposed that animals use phages as weapons against infections, alongside established humoral and cellular immunity. At the same time, Mechnikov speculated that phagocytes were simple cells in animals that fought foreign bodies, a perspective similar to d’Hérelle’s view on bacteriophages. Mechnikov described phages as neither viruses nor enzymes but dried-out bacteria capable of passing through bacteriological filters. Leopold Perets, a Leningrad microbiologist, expanded Mechnikov’s theory, suggesting that phages cause infections by killing beneficial bacteria (*E. coli*). Moscow microbiologist Lev Zilber, in his 1928 monograph Para-Immunity Against Microbes, supported d’Hérelle’s phage model. Magdalena Pokrovskaya, from the Anti-Plague Institute in Stavropol, studied the role of phages in bubonic plague, isolating plague bacteriophages from the bodies of dead rodents [41,42].

#### 1.2.2. Bacteriophage Research History in Bulgaria

Bulgaria has a long-standing history in bacteriophage research, with early efforts centered on exploring their lytic activity and potential in bacterial control. A notable early study in this field was carried out in 1969 by the military doctor Vladimir Kasovski [11], who focused on isolating, characterizing, and applying bacteriophages against *Bacillus* species—specifically *Bacillus anthracis*, the pathogen responsible for anthrax. Using classical enrichment and plaque assay methods, particularly the double-layer agar technique, Kasovski isolated lytic phages from environmental sites linked to infected animals. The research included host range testing and preliminary morphological assessments to determine the phages’ specificity and lytic strength, though it lacked electron microscopy images. The study aimed to identify phages with strong specificity toward *B. anthracis*, assess their stability in various environmental conditions, and explore their potential use in diagnostics. Several phages were successfully isolated, showing potent lytic activity against multiple *B. anthracis* strains while exhibiting limited or no effect on other *Bacillus* species [11].

**Figure 2:**
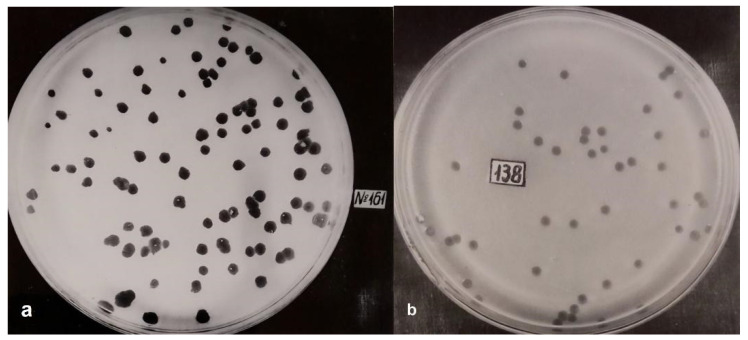
Morphological analysis of *B. anthracis* phage plaques demonstrating host-specific lytic activity. (**a**): Plaques (“negative colonies”) produced by phage 161 on *B. anthracis* strain 34F. The plaques demonstrate the specific lytic activity of the phage, as shown by the clear plaques indicative of bacterial lysis. (**b**): Plaques (“negative colonies”) produced by phage 138 on *B. anthracis* strain Davis. The distinct plaque morphology underscores the phage’s ability to lyse the bacterial strain, confirming its host specificity and bactericidal efficacy. Image obtained from Kasovski, 1969s [11].

The plaque morphology observations (Figure 2) revealed clear lytic activity by the isolated bacteriophages against different *B. anthracis* strains. Figure 2a shows plaque formation (“negative colonies”) produced by phage 161 on *B. anthracis* strain 34F, indicating successful infection and localized bacterial lysis. Figure 2b demonstrates more extensive lysis by phage 138 on strain Davis, suggesting stronger infectivity and broader lytic activity against this host strain. These plaques indicated efficient infection and bacterial lysis, with no evidence of temperate or lysogenic behavior. However, in the absence of molecular tools, lysogeny could not be definitively ruled out. While the study predated modern genomic and structural biology techniques, it included thermal and pH stability testing, showing that phages remained viable at 4 °C over extended periods, with infectivity maintained up to approximately 55 °C and in a neutral pH range. This early assessment of environmental resilience was a strength of the work and remains relevant in the context of phage storage and transport. The effective lysis of *B. anthracis* strains in this study supports the potential for phage-based bacterial control. The results highlighted the strain-specific nature of bacteriophage activity, with some phages demonstrating highly lytic behavior against certain species, while others exhibited limited or no infectivity. Such narrow host range underscored their potential use in diagnostic phage typing schemes for *B. anthracis* identification during outbreak investigations.

Despite these contributions, several limitations are evident when viewed through a modern lens. While electron microscopy was employed in the study to document phage morphology, the work however, did not incorporate more advanced molecular techniques such as sequencing, PCR-based characterization, or blotting methods. The lack of these approaches hinders the possibility to determine genomic composition, confirm phage identity and purity at the molecular level and compare these isolated phages with current phage databases. Since genetic features of the phages have strong influence on factors such as host range, environmental stability, and therapeutic potential, the lack of molecular characterization in this study ultimately restricts the relevance of the study to current taxonomic standards and its applicability in contemporary phage therapy research.

The lack of genetic characterization left uncertainty as to whether these phages were strictly lytic (suitable for therapeutic use) or lysogenic (which could pose risks in medical applications due to gene transfer potential). Additionally, as whole-genome sequencing, PCR, or receptor-binding analysis were not available at the time, accurate classification and assessing the long-term stability of these phages is not possible. Moreover, the study did not investigate phage efficacy against biofilms or consider formulation strategies for pharmaceutical use which are critical factors in treating chronic bacterial infections today. This highlights the need to re-examine historical phage isolates using current molecular and translational tools.

Another significant limitation is the lack of cytotoxicity assessment, a crucial aspect of modern phage therapy research. While bacteriophages are generally considered non-cytotoxic, evaluating their interaction with human cells and immune responses, through in vivo immunogenicity assessments is necessary to validate their safety for therapeutic applications. This leaves questions about therapeutic safety and host immune responses unanswered. Additionally, the study did not explore the impact of formulation additives or stabilizers, which are essential for ensuring the stability and efficacy of phage-based treatments. Although the study did not explore therapeutic applications directly, it laid important groundwork for phage-based diagnostics in anthrax surveillance and represents a notable milestone in the development of Bulgarian bacteriophage research.

Although limited by the technological capabilities of its time, the study’s historical value remains significant. It established a foundational understanding of host specificity, bacterial susceptibility, and lytic activity-concepts central to modern bacteriophage therapy. In this light, although not directly related to Dr. Kasovski’s work, very recent research has pinpointed a therapeutic promise of *Staphylococcus*-specific phages like *Staphylococcus* phage SDQ in managing MRSA and skin infections [43]. Thus, revisiting early Bulgarian work using modern techniques such as whole genome sequencing, cytotoxicity profiling, and advanced phage-host interaction models, could uncover valuable therapeutic insights for the future.

In 1973, a comprehensive study conducted by Krachmarova-Raycheva focused on the isolation and characterization of bacteriophages targeting *K. pneumoniae* and *Enterobacter aerogenes*, now *Klebsiella aerogenes* [12]. Using a modified version of Aldová’s enrichment method [44] (Table 2 showing comparison between the original and modified protocols), adapted to clinical isolates, selective host strains, and optimized incubation conditions, the researchers successfully isolated 13 bacteriophages, 11 of which were specific to *K. pneumoniae* and two to *E. aerogenes*. These phages, recovered from canal water and fecal samples, were classified into distinct groups based on their lytic activity, host specificity, and plaque morphology. The use of clinical strains and phenotypic classification schemes was a notable strength, increasing the medical relevance of the findings. However, Aldová’s method is fully culture-dependent and may fail to detect phages that require more specific propagation conditions or exhibit atypical lysis. Additionally, it does not permit genomic characterization, limiting confirmation of whether the phages were strictly lytic or potentially temperate; a critical distinction in therapeutic contexts. The researchers further enhanced their approach by incorporating phage stability and thermal tolerance testing, offering valuable insights into phage viability for potential medical use.

**Figure 3:**
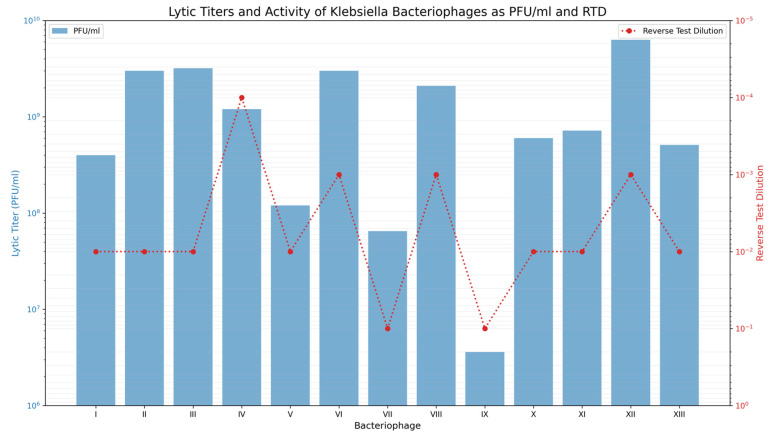
Lytic titers and reverse test dilutions (RTD) of *Klebsiella* bacteriophages. The bar chart presents lytic titers (PFU/mL), quantifies the number of infectious phage particles detected by plaque formation, alongside reverse test dilution (RTD) values (expressed as negative powers of 10) that indicate the highest dilution at which phage lytic activity remained detectable in the liquid Appleman’s test.

**Figure 4:**
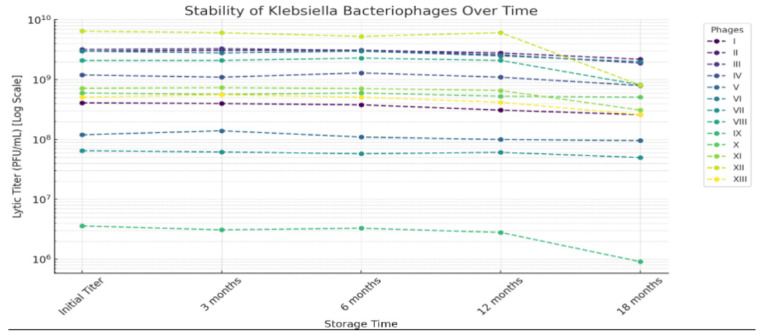
Stability of *Klebsiella* bacteriophages over time: This graph represents the stability of *Klebsiella* bacteriophages over time, illustrating changes in lytic titers (PFU/mL) at different storage periods (3, 6, 12, and 18 months). The numerical values are based on the results obtained in the study and are presented in a broader manner to convey the overall trends in phage stability. The graph highlights the persistence of phage activity over time, with some decline observed at later time points, which is crucial for understanding their potential application in medical and therapeutic settings.

The lytic titers and reverse test dilution (RTD) values reported for the phages against *Klebsiella* are summarized in Figure 3. The lytic titers, measured using Gratia’s double-agar-layer method, reflect the concentration of infectious phage particles capable of forming plaques on a susceptible host. On the other hand, the RTD values indicate the highest dilution at which each phage still demonstrated measurable lytic activity in the liquid Appleman’s assay, with lower RTD values representing greater apparent potency. It is crucial to note that both parameters are hugely influenced by confounding factors such as host strain used, receptor compatibility, assay methodology, and growth conditions. Consequently, the reported values should be interpreted as products of these experimental conditions rather than as definitive indicators of inherent phage potency. The study lacked in vivo validation or cytotoxicity assessments that limited its immediate translational potential. Further stability testing, presented in Figure 4, demonstrated that most phages remained viable for up to 18 months, although gradual declines in infectivity were observed. Several phages also retained activity at temperatures up to 60 °C, with significant inactivation beyond 65 °C. These results have practical implications for storage and transport in clinical settings. However, the study did not investigate the use of stabilizing agents or pharmaceutical formulations—critical for modern therapeutic applications.

The researchers adapted Aldová’s classic phage isolation technique to a clinical context by using patient-derived isolates of *K. pneumoniae* and *E. aerogenes* as both sources and indicator hosts. They optimized incubation conditions and included a post-isolation phage typing scheme to assess host range across strains from various hospitals, which was not part of Aldová’s original protocol. These methodological enhancements increased the likelihood of isolating clinically relevant phages and enabled strain-level epidemiological tracking.

**Figure 5:**
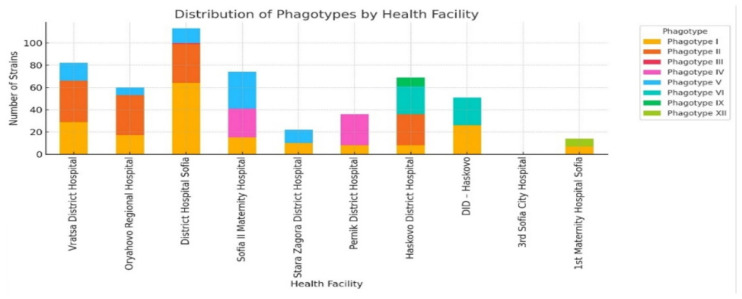
Distribution of phagotypes of *K. pneumoniae* strains by health facility, based on data from Krachmarova-Raycheva’s 1973 study [12].

A series of assays evaluating the interaction between the isolated *K. pneumoniae* phages and a panel of bacterial species demonstrated that most of the phages exhibit high host specificity. In these tests, the phages showed effective lytic activity against *K. pneumoniae* while producing little to no lytic effect on other bacteria such as *E. coli*, *Salmonella*, *Shigella*, *Proteus*, and *Pseudomonas* spp. Notably, phages VIII and IX demonstrated slightly broader host ranges, suggesting potential for wider therapeutic applications. While high specificity is advantageous for preserving the microbiota, it may limit the use of these phages in polymicrobial infections unless formulated into cocktails. Electron microscopy was reportedly used to observe phage morphology, aiding in classification, but no visual documentation was provided, restricting structural comparisons with modern phages, where morphology informs host interaction and stability. Furthermore, although some phages showed efficacy against antibiotic-resistant *K. pneumoniae* strains, the absence of immunogenicity and cytotoxicity data leaves key safety questions unanswered. Without molecular confirmation or host immune response studies, it remains unclear whether these phages could be safely translated into therapeutic use. Beyond therapeutic potential, the study contributed to early phage typing for epidemiological purposes. It achieved a typability rate of 86.64% in *K. pneumoniae* strains and revealed the widespread distribution of Phage Groups I and II across multiple hospitals. Figure 5 visualizes this distribution, reinforcing the reproducibility and epidemiological significance of the phage typing scheme. However, while valuable for outbreak tracking, the study did not explore the application of these phages in infection control, missing an opportunity to link epidemiology with intervention.

The phage types (I–XII) among *K. pneumoniae* strains were isolated from various Bulgarian health facilities. Clinical isolates were collected from the hospitals and submitted for centralized phage typing at the Institute of Microbiology, Bulgarian Academy of Sciences. Phage types I and II appear most frequently across multiple sites, suggesting their widespread presence and possible association with healthcare-related transmission. These data highlight the epidemiological significance and reproducibility of the phage typing scheme used in the study, which provided an early method for distinguishing *K. pneumoniae* strains and identifying patterns that could be linked to potential hospital outbreaks. The figure reflects the diversity and spread of specific phage types within various healthcare settings, supporting the findings of the study and its impact on phage typing and outbreak tracking. While the variation in phagotype prevalence by hospital is shown here, the contextual information about the facilities themselves (such as size, specialization, or patient population) were no provided, limiting deeper epidemiological interpretation.

Despite its limitations, the 1973 study remains a foundational milestone in Bulgarian bacteriophage research. Its contributions to early phage isolation, host specificity profiling, stability testing, and epidemiological surveillance laid important groundwork for understanding *K. pneumoniae* phage biology. Yet, the absence of genomic sequencing, lysogeny screening, cytotoxicity data, and in vivo testing limits the study’s relevance to current therapeutic standards. The lack of contextual information about the healthcare facilities involved, such as size, specialization, or patient demographics further constrains epidemiological interpretation, making it difficult to assess why certain phagotypes were more prevalent in specific settings. Additionally, the study did not assess phage lifecycle parameters like burst size or latency, nor did it evaluate the impact of formulation additives or stabilizers that are critical for modern pharmaceutical development. Without visual documentation from electron microscopy or data on immunogenicity, structural classification and host safety remain uncertain. Taken together, these gaps highlight the importance of reassessing this work using modern tools such as whole-genome sequencing, cytotoxicity assays, and clinical validation. As Bulgaria renews its investment in phage research, revisiting this historic study through the lens of contemporary genomics, immunology, and infection control may unlock new potential for combating antibiotic-resistant *K. pneumoniae*.

A later study in 1976 by Paparkova focused on *Enterococcus* bacteriophages, further expanding Bulgaria’s contributions to phage biology [45]. Using Aldová’s isolation method—previously modified by Krachmarova in 1973 [12], the researchers refined techniques to improve phage recovery from complex environments. The study also incorporated the critical test dilution method, previously evaluated by Steyo and Chep [46], to quantify phage-host interactions and lytic efficiency. However, no detailed explanation of these methodological modifications was provided, leaving uncertainties about their specific impact on isolation outcomes.

A total of 18 bacteriophages were successfully isolated from clinical and environmental sources, including wastewater and hospital effluents. These phages were classified into two major serological groups, each with four subgroups, based on antigenic properties and host range specificity. While this serological approach provided an early framework for phage classification, the absence of molecular validation limits its taxonomic accuracy. In modern research, genomic sequencing and proteomics are standard tools for phage characterization and serotyping tools that were unavailable at the time make the classification system relatively imprecise by current standards.

**Figure 6:**
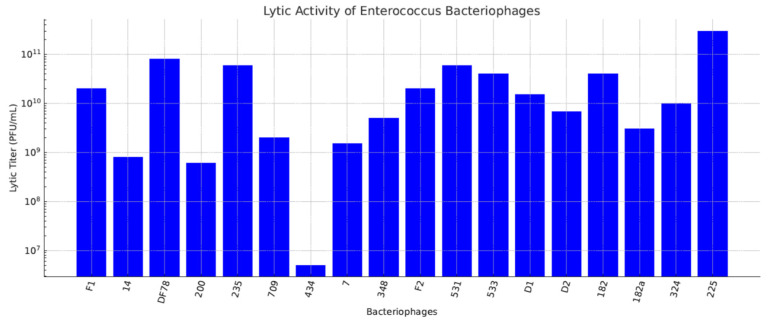
Lytic activity of *Enterococcus* bacteriophages on individual host strains. This bar chart presents the lytic titers (PFU/mL) of 18 *Enterococcus* bacteriophages described in Paparkova’s 1976 study [45]. The phages were tested against individual hosts which were different strains of *Enterococci* bacteria. No clarifications on the number or identity of the bacterial strains, nor on the bacteriophages used against each strain were provided. The data were extracted from the study and visualized to illustrate the wide lytic activity of the phages against *Enterococcus* strains.

Figure 6 illustrates the lytic titers obtained for 18 *Enterococcus* bacteriophages described in Paparkova’s 1976 study [45]. Each phage was evaluated using its corresponding *Enterococcus* host strain, although the number and identity of the bacterial hosts used for these assays were not reported. This lack of methodological detail limits direct comparison of host specificity or relative potency across the phage set. As with the *Klebsiella* data (Figure 3), the reported titers should be interpreted cautiously, as they are shaped by assay conditions, including host–phage compatibility, the physiological state of the host culture, and the specific methods employed. The figure nonetheless highlights the considerable variation in measured lytic activity among the isolates. Furthermore, the study did not determine whether these phages were strictly lytic or temperate, a critical consideration in therapeutic safety, as lysogenic phages may carry genes that promote antibiotic resistance or bacterial virulence. Moreover, although the study points out that the *Enterococcus* bacteriophages were tested against individual host strains rather than a single common host, the total number and the specific strain of the *Enterococcus* tested by each phage is not clarified. This limitation diminishes the possibility to compare host ranges across the full set of phages.

**Figure 7:**
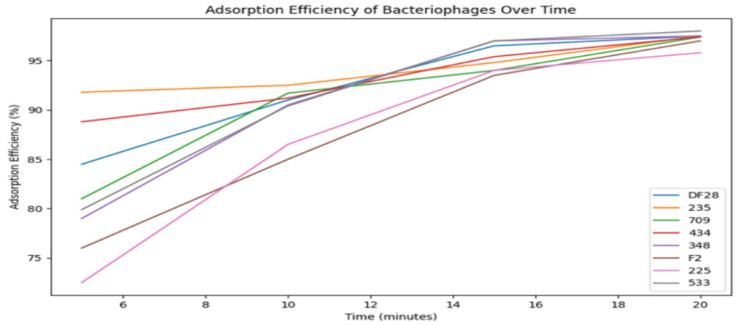
Adsorption efficiency of enterococcal bacteriophages on individual host strains. The percentage of bacteriophages adsorbed to bacterial cells was measured at different time points (5, 10, 15, and 20 min). The results were obtained from and transformed into a graphical representation for improved visualization. The data show that most bacteriophages exhibit rapid adsorption within the first 10 min, with adsorption efficiencies reaching between 85% and 98% by the 20 min mark. Phages 225 and F2 showed a slightly slower initial adsorption rate but eventually achieved high efficiency. This visualization highlights the fast and efficient adsorption kinetics of enterococcal bacteriophages.

Further adsorption efficiency analysis, presented in Figure 7, showed that most bacteriophages exhibited rapid adsorption, with rates between 85% and 98% within 20 min. High adsorption rates imply strong host-binding capabilities and effective infection dynamics. However, the study did not explore receptor-binding mechanisms, which would be essential to understanding how these phages recognize and attach to bacterial surface receptors. Notably, phages 225 and F2 demonstrated slower initial adsorption but eventually reached high efficiency, suggesting variability in host recognition dynamics.

**Figure 8:**
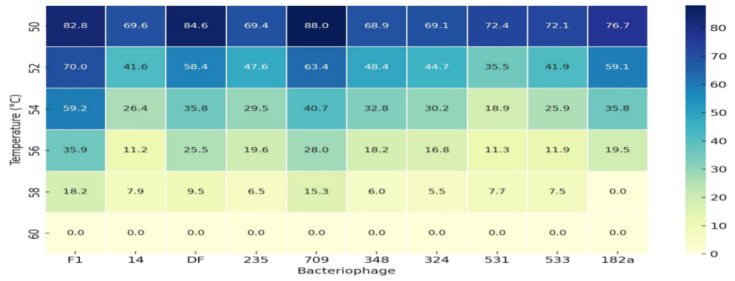
Heatmap of thermal inactivation of *Enterococcus* bacteriophages at different temperatures. The heatmap represents the percentage of surviving phages after exposure to various temperatures (50–60 °C) for 10 min. The data were extracted from a table provided in the study paper and transformed into a heatmap for improved visualization. Darker shades indicate higher survival rates, while lighter shades indicate increased inactivation at higher temperatures. Most phages exhibit stability up to 50 °C but show significant inactivation at 60 °C.

Phage replication kinetics revealed latent periods ranging from 12 to 32 min and burst sizes of 91 to 120 virions per infected cell—values consistent with efficient lytic phages. While these parameters reinforce their potential for bacterial control, the absence of any in vivo testing limits conclusions about real world efficacy in treating *Enterococcus* infections. Thermal stability results, summarized in Figure 8, showed that most phages remained active up to 50 °C, with substantial inactivation at 60 °C. This indicates moderate heat tolerance, a useful trait for pharmaceutical preparation. However, the study did not assess long-term viability under refrigeration or the effects of stabilizing additives, key factors in the formulation and storage of therapeutic phages. Host range analysis confirmed that these phages were highly specific to *Enterococcus* strains, supporting their potential use in precision-targeted therapy. However, this narrow specificity also restricts their utility in polymicrobial infections unless used as part of a phage cocktail. This limitation remains relevant in modern phage therapy, where combining multiple phages is often necessary to broaden host range and reduce resistance development.

While the study provided valuable insights into phage stability, adsorption, and lytic potential, it also had notable limitations. It did not explore activity against antibiotic-resistant *Enterococcus* strains, such as vancomycin-resistant *Enterococcus* (VRE), which are of high clinical concern today. Additionally, no cytotoxicity testing was performed, leaving the immunological safety of these phages undetermined. Without genomic analysis, there is also no way to confirm the absence of lysogenic or virulence-related genes. Most significantly, the lack of animal or clinical studies prevents conclusions about therapeutic effectiveness in vivo.

Despite these limitations, Paparkova’s 1976 study remains a foundational contribution to Bulgarian phage research [45], particularly in the systematic isolation and early classification of *Enterococcus* specific phages. However, its lack of molecular and translational data highlights the need for modern reassessment. Revisiting these isolates with genomic sequencing, safety profiling, and in vivo efficacy studies could determine whether they have relevance in today’s therapeutic landscape. With growing interest in phage therapy worldwide, this early work provides a valuable historical benchmark for Bulgaria’s evolving role in bacteriophage science.

Around the same period, Karaivanov (1976) conducted a notable study on the physical and chemical stability of *Pasteurella multocida*-specific bacteriophages, offering a rare publicly accessible example of Bulgaria’s early phage research [47]. Unlike Paparkova’s more technically elaborate work, which is available only through limited institutional archives, Karaivanov’s study accessible through public scientific databases, focused on phage resilience to environmental and chemical stressors, and has received relatively wider recognition for offering a clearer glimpse into early Bulgarian phage research. Most phages showed progressive inactivation from 48 °C, with up to 99.99% loss at 65 °C, though group III phages displayed delayed inactivation between 50 °C and 60 °C. Optimal survival occurred at pH 7.5, with group I phages being most sensitive to deviations. Chemically, potassium permanganate and pyronin inactivated 90–100% of phages, while urea and sodium citrate allowed partial survival in some cases [47]. While robust in methodology, the study lacks molecular or host range data and provides no safety assessment, key gaps by current standards. Still, its early exploration of phage robustness remains relevant today, particularly for therapeutic formulation and stability. The availability of this study presents a valuable opportunity to revisit its findings with modern tools, potentially reclaiming overlooked phages for use against antibiotic-resistant pathogens.

Taken together with Paparkova’s contemporaneous work, Karaivanov’s study highlights the diverse research directions pursued in Bulgaria during this early period of phage science. While Paparkova focused on biological function and host interaction, Karaivanov addressed environmental durability, each contributing distinct yet complementary insights. Their combined findings, though methodologically limited by the era’s tools, underscore an early awareness of both therapeutic potential and practical challenges in phage application. This dual perspective forms a valuable foundation for modern reassessment, where stability and efficacy must go hand in hand in developing phage-based solutions to antibiotic resistance.

Extending the scope of the earlier work, Savov et al. (1977) (work available through public databases hence offering broad insight into applied potential of early Bulgarian phage research), investigated lysogeny in *E. coli* strains isolated from birds, adding a new ecological dimension to Bulgaria’s early phage studies [48]. Out of 70 avian *E. coli* isolates, 19 lysogenic strains were identified, and the resulting phages were grouped into two serologic categories based on neutralization assays. Representative phages demonstrated cross-species lytic activity, not only lysing bird-derived *E. coli* strains but also showing limited activity against isolates from calves and pigs, as well as select *Salmonella* strains. This study moved beyond laboratory characterization to propose practical applications for phages in tracing infection sources and evaluating disinfection strategies, reflecting an early awareness of phages as tools for epidemiological surveillance. Compared to the 1976 studies, which focused separately on biological behavior (Paparkova, 1976 [45]) and environmental resilience (Karaivanov, 1976 [47]), this research integrated aspects of host ecology and interspecies transmission, suggesting broader implications for phage utility in veterinary and public health contexts. Nonetheless, like its predecessors, it lacked genomic validation, therapeutic safety testing, or investigation into lysogenic phages’ potential to carry virulence genes, an especially important consideration given their application in infection tracking. Despite these gaps, the study advanced Bulgaria’s phage research by linking basic virology with applied epidemiology, a step forward in recognizing the multifaceted potential of bacteriophages.

Later in 1980, further building on earlier bacteriophage research in Bulgaria, Trifonova and Bratoeva conducted a study focused on the intraspecific differentiation of *Shigella sonnei* strains by integrating a broader range of microbiological techniques [49]. While Paparkova, Karaivanov, and Savov et al. [45,47,48] had focused primarily on phage isolation, host range studies, and physical stability, Trifonova and Bratoeva advanced the approach by combining phage typing with biochemical profiling such as carbohydrate fermentation and enzymatic activity tests [49,50], and serological typing based on surface antigen differences [51]. This multi-method strategy enabled finer discrimination between closely related *S. sonnei* strains, improving the precision of epidemiological surveillance. Conducted in Bulgaria but published in Russian, their study emphasized the practical application of phages not only as biological tools but also as critical components of infectious disease management. Unlike some contemporary works available only through institutional archives, this study remains accessible through public databases, offering broader visibility. Compared to the earlier 1976–1977 studies, which primarily explored phage biology in controlled laboratory settings, Trifonova and Bratoeva’s work marked a significant shift toward operational, field-based applications of phage science [49]. However, consistent with the limitations of the era, the study lacked molecular or genomic validation and did not address potential genetic risks associated with lysogenic phages. Nevertheless, it expanded the role of phages in Bulgarian microbiological research, demonstrating their potential beyond therapeutic exploration toward epidemiological tracking and infection control.

The first studies related to bacteriophages and *Salmonella* date back to 1975 [52] and 1987 [13]. A study by Mollov expanded earlier Bulgarian research by focusing on nine bacteriophages specific to *Salmonella enteritidis* [13]. The aim was to elaborate a phage typing scheme, in order to be employed for diagnostic purposes and used for epidemic studies, as well as, to evaluate the phages’ lytic activity, host range, lysogenic behavior and stability. These phages were isolated from environmental and clinical samples and tested on *S. enteritidis* strains to determine their lytic capacity and typability.

**Figure 9:**
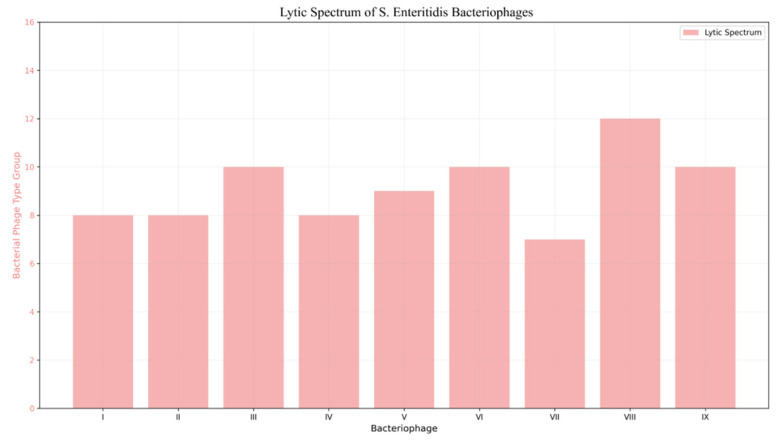
Lytic spectrum of *Salmonella enteritidis* bacteriophages. The bars show the lytic spectra of nine *S. enteritidis* bacteriophages (I–IX) reflecting the total number of phage groups susceptible to a particular phage.

A total of 1083 *S. enteritidis* strains were evaluated and organized into 15 lytic susceptibility groups. The lytic spectrum analysis revealed considerable diversity: Phages III, VI, VIII, and IX exhibited the broadest host ranges, lysing the highest number of bacterial groups. Notably, Phage VIII lysed 12 groups, demonstrating the widest host range within the phage collection (Figure 9). These findings seem to indicate significant variation in potency and host specificity among the early Bulgarian *S. enteritidis* bacteriophages.

The study also evaluated both lytic and lysogenic activity of the phages. Some, like Phages VII and IX, exhibited moderate levels of lysogeny (with lysogenization percentages ranging from 71–84%), though not all displayed this trait strongly. Meanwhile, others showed predominantly lytic activity.

Distinction between lytic and lysogenic activity of phages is crucial for understanding the dual nature of bacteriophage action, especially in therapeutic contexts, where lytic-only phages are generally preferred due to the risks associated with lysogeny, such as the potential transfer of antibiotic resistance or virulence genes. The absence of molecular screening means lysogeny cannot be conclusively ruled out in any phage.

**Figure 10:**
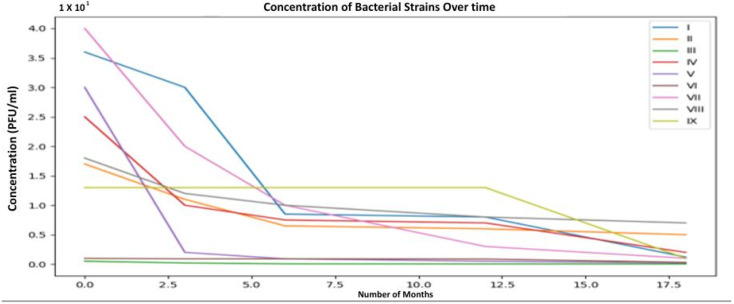
Stability of *S. enteritidis* bacteriophage strains over 18 months.

Phage stability was evaluated by monitoring titer changes over 18 months of storage. Figure 10 demonstrates that while some phages showed a moderate decline in activity, most retained reasonable stability over time. However, the storage conditions were not specified, and no stabilizers were tested, making it difficult to assess their pharmaceutical readiness. Stability in varying storage temperatures, particularly under refrigeration or freeze-dried formats, would have been critical to determine long-term viability for clinical use.

This graph illustrates the changes in concentration (measured in PFU/mL) of nine *S. enteritidis* bacteriophage strains (labeled I to IX) over an 18-month period. The data tracks the stability of the phages at various time points: 0 months (initial concentration), 2.5 months, 5 months, 10 months, and 17.5 months. Each line represents a different bacteriophage strain, with the *x*-axis showing the number of months and the *y*-axis showing the phage concentration. The graph highlights the variation in stability and persistence of the phages over time, with some strains maintaining higher concentrations while others show a significant decline. These data are essential for understanding the long-term effectiveness of these bacteriophages in applications like phage therapy and environmental control.

Although the study contributed important data on phage activity, stability, and lysogeny, it lacked molecular analysis to determine whether the phages carried lysogenic or virulence-associated genes. It also did not assess their activity against antibiotic-resistant *Salmonella* strains, which limits its relevance to current therapeutic challenges. Additionally, no cytotoxicity or immunogenicity tests were conducted, which are essential to determine host safety. The absence of in vivo experiments further limits the clinical translation of these findings. Nonetheless, this study provides an early and detailed framework for phage-based differentiation of *S. enteritidis* strains, offering potential diagnostic utility. Reassessing these isolates using genomic tools, receptor-binding studies, and in vivo models could uncover their potential for both therapeutic and diagnostic applications. With renewed global interest in phage therapy, revisiting such foundational studies offers an opportunity to uncover underutilized resources for combating bacterial infections.

Following a long gap in Bulgarian research, a pivotal study published by Ishlimova et al. in 2009 investigated the genetic diversity of bacteriophages infecting *Streptococcus thermophilus* strain LBB.A, a well-established component of traditional Bulgarian yogurt cultures [53]. Eight virulent phages were isolated, all displaying extremely narrow host ranges, primarily infecting only LBB.A. Interestingly, while host specificity was consistent, genetic analysis revealed substantial diversity among the phages, with at least five being genetically unrelated despite all belonging to the cos-type group. A key strength of the study lies in its practical relevance to the dairy industry, where phage infections pose ongoing risks to fermentation reliability and product quality. The research employed a comprehensive set of classical and molecular techniques: restriction digestion, PCR, pulsed-field gel electrophoresis, and host range assays strengthening the validity of its findings. Notably, the development of phage-insensitive mutants through a straightforward two-step challenge method, without compromising acidification performance, provided a viable solution for industrial application.

Building on this foundational work, a later study in 2012 again led by Ishlimova and colleagues expanded the investigation to include the broader diversity of bacteriophages infecting the *S. thermophilus* component of various industrial yogurt starters in Bulgaria [54]. This study focused on phage diversity and host specificity, offering insights not only into food microbiology but also into potential medical applications, particularly in the context of antibiotic resistance. The study’s use of phage isolation, host range determination, and genetic characterization aligned closely with techniques employed in therapeutic phage research. The identification of highly specific lytic phages against *S. thermophilus* reinforces the feasibility of targeting pathogenic bacteria with minimal collateral damage—a critical consideration in clinical applications. Their findings regarding narrow host specificity are particularly relevant when selecting phages to combat multidrug-resistant (MDR) pathogens without disrupting beneficial microbiota. However, this same specificity also presents a challenge: the need for broader host-range phages or phage cocktails to ensure therapeutic efficacy. Moreover, the use of restriction fragment length polymorphism (RFLP) profiling to differentiate phage strains could be translated into clinical practice for monitoring phage adaptation and resistance evolution. The discovery of two distinct phage types, cos- and pac- types, suggests that understanding packaging mechanisms may help develop genetically stable phage therapies less prone to mutation-driven failure.

Both studies share several methodological strengths and limitations that are important to highlight. On the one hand, they demonstrate consistent application of classical and molecular techniques, including host range assays, PCR, and RFLP profiling, all of which provide valuable insights into phage diversity and specificity. Their identification of highly specific lytic phages supports the concept of targeted bacterial control in both food and clinical microbiology. On the other hand, both studies lack whole-genome sequencing, which limits deeper genetic insight, including the ability to detect virulence genes or antibiotic resistance elements. The proposed involvement of CRISPR (Clustered Regularly Interspaced Short Palindromic Repeats), a bacterial adaptive immune system that protects against phage infection by recognizing and cleaving foreign DNA [55], in resistance mechanisms is mentioned but not experimentally validated in either study. This absence of direct experimental confirmation weakens the strength of the conclusions regarding resistance, as interpretations remain speculative and the precise role of CRISPR in phage-host dynamics cannot be confidently established. Consequently, this limits both the mechanistic understanding of resistance and the reliability of potential industrial applications. Additionally, neither includes environmental or production-scale testing, which limits the understanding of phage performance under real-world conditions.

Despite these shared gaps, the work of Ishlimova et al. underscores Bulgaria’s potential in advancing phage research beyond food microbiology. By applying similar phage isolation and characterization techniques to clinical pathogens, researchers can explore phage therapy against antibiotic-resistant infections, a crucial step in addressing the global antimicrobial resistance crisis [53,54].

Later in 2013, Aleksandrova et al. conducted a study to classify the newly isolated bacteriophage φGb1, which infects *Lactobacillus delbrueckii* ssp. *bulgaricus*, marking the first report of a Group “B” *L. bulgaricus* phage in Bulgaria [56]. Unlike earlier Bulgarian studies from 2009 and 2012, which lacked genome-level data, this research incorporated partial genome sequencing of 31 cloned DNA fragments, enabling the classification of φGb1 based on high similarity (80–99%) to known Group “B” phages LL-Ku and c5. This genetic insight represents a methodological advancement over the prior studies, which focused more on host range and phenotypic diversity without sequencing. However, φGb1’s host specificity, infectivity, and ecological behavior remain unexplored, and no morphological or functional characterization was conducted, contrasting with the broader biological assessments in the earlier work. While still not a full genome sequence, the molecular data lay the groundwork for future genomic and therapeutic research. Importantly, this study reinforces the value of phage classification and genetic precision critical considerations in medical microbiology, where understanding phage-host interactions, genomic safety, and specificity is essential for developing bacteriophage-based therapies against antibiotic-resistant pathogens. Although φGb1 was isolated from a food microbiology context, its targeted nature and minimal genetic overlap with unrelated hosts support its potential for safe application with reduced risk of cytotoxicity or off-target effects in therapeutic settings.

Demonstrating the evolving versatility of bacteriophage research in Bulgaria, a study by Kizheva et al. (2021) focused on phages isolated from the rhizosphere of healthy tomato plants, aiming at plant disease control but offering insights highly relevant to medical microbiology [57]. The researchers investigated broad-host-range lytic phages targeting phytopathogens such as *Xanthomonas* spp. Although the study was primarily aimed at controlling bacterial spot disease in crops, the isolation of strictly lytic Podovirus-like phages capable of infecting multiple *Xanthomonas* strains mirrors principles used in therapeutic phage cocktail development. The authors evaluated phage resilience under thermal stress, UV exposure, and storage conditions, factors critical to the stability and viability of phages intended for clinical use. Compared to earlier Bulgarian studies that focused on dairy-associated *S. thermophilus* or *L. bulgaricus*, this work introduced environmental sampling and broader host-range assessments, aligning more closely with the requirements for medical phage applications targeting multidrug-resistant bacteria.

Like earlier studies, however, this research was limited by the absence of whole-genome sequencing, leaving crucial safety concerns unaddressed, particularly the potential presence of lysogeny-related genes, virulence factors, or antimicrobial resistance elements. Furthermore, the study did not evaluate cytotoxicity or immune activation in mammalian systems, which are critical considerations before advancing toward clinical applications. The phages were tested solely on plant pathogens, and their cross-reactivity with medically relevant Gram-negative bacteria, such as *P. aeruginosa* or *E. coli*, remains unknown. Nonetheless, the study underscores the feasibility of leveraging broad-host-range, stable, and lytic phages for medical applications—provided that future work addresses these safety and specificity gaps.

Later, a more recent study by Kizheva et al. (2023) conducted a detailed laboratory-based investigation characterizing 11 newly isolated bacteriophages targeting *Xanthomonas euvesicatoria*, a major plant pathogen [58]. Their work focused on phenotypic and genotypic analysis, including plaque morphology, host range profiling, electron microscopy, and partial genome characterization. Among the isolates, phage BsXeu269p/3 emerged as particularly promising due to its broad host activity within *X. euvesicatoria* strains, stability under diverse environmental conditions, and lack of adverse effects on beneficial rhizosphere bacteria. In a subsequent follow-up, Shopova et al. (2023) focused specifically on this phage, evaluating its biocontrol potential through in vivo greenhouse trials on pepper plants [58]. Their study demonstrated a significant reduction in disease incidence, highlighting its practical relevance in agricultural settings. Furthermore, the use of qPCR to track phage and pathogen populations, along with persistence assessments in plant tissues, introduced methodological approaches that could inform applications in medical microbiology. Despite these strengths, both studies share limitations, notably the absence of whole-genome sequencing and the lack of evaluation regarding phage cytotoxicity, immune response, or resistance development—critical parameters for both agricultural and clinical deployment. While Kizheva et al. provide a comprehensive in vitro foundation for candidate phage selection, Shopova et al. offer clearer evidence of real-world efficacy. Taken together, the two studies represent sequential steps in the development of phage-based solutions, though future research would benefit from integrating genomic, ecological, and translational considerations to fully realize their potential.

Together, these more recent studies show Bulgaria’s continued contribution to phage research, expanding beyond dairy associated microbiota into agricultural and potentially medical applications. While not initially designed for therapeutic use, the methodologies and findings offer a solid foundation for adapting bacteriophages to clinical microbiology. To fully realize this potential, future Bulgarian research must integrate comprehensive genomic safety profiling, host-range broadening, cytotoxicity testing, and exploration of optimized delivery systems. Such advances would enable the development of phage-based therapies capable of addressing antibiotic resistance and minimizing cytotoxic risks, a key priority in modern medical microbiology.

### 1.3. Current Trends and Future Directions

#### 1.3.1. Recent Research Trends in Bacteriophage Application

Advanced in vitro models using mechanism-based approaches have further refined our understanding of phage pharmacodynamics, enabling optimization of dosing regimens for phage cocktails to maximize bacterial killing while minimizing resistance development [59]. In parallel, recent studies have underscored the safety of bacteriophages in eukaryotic systems. A review from 2025 demonstrated the synergistic potential of combining bacteriophages with mesenchymal stem cells (MSCs) in therapeutic contexts such as wound healing, with no observed cytotoxicity on MSCs [16]. Moreover, primary research employing advanced high-content imaging techniques has shown that bacteriophages can interact with epithelial cells without inducing direct injury or cytotoxic effects [60]. Complementing these mechanistic insights, a Phase 2 clinical trial published in 2024 reported that combining a phage cocktail with the antibiotic Bactrim resulted in a rapid reduction in *E. coli* levels in patients with urinary tract infections, reinforcing the potential for synergistic treatment modalities [61]. Together, these findings highlight the potential of bacteriophages and their engineered variants as safe, non-cytotoxic agents in the fight against antibiotic-resistant bacteria, while also emphasizing the necessity for further research to define their safety across varying concentrations.

#### 1.3.2. Global Advancements in Bacteriophage Research: The Contrast with Bulgaria

While historical bacteriophage research in Bulgaria laid the foundation for understanding phage-host interactions and potential medical applications, recent global advancements have propelled phage therapy into an era of clinical translation and technological innovation. A 2024 study published in Nature Microbiology analyzed 100 individualized phage therapy cases across 12 countries, demonstrating clinical improvement in 77.2% of patients and bacterial eradication in 61.3%, particularly when combined with antibiotics [62]. Similarly, a large-scale trial on urinary tract infections (UTIs) caused by *E. coli* has established dosing regimens for future trials, pushing phage therapy closer to standardized medical use [63]. Meanwhile, Locus Biosciences’ pioneering work on CRISPR-enhanced bacteriophages has yielded promising results in the targeted elimination of antibiotic-resistant pathogens, reinforcing the therapeutic potential of engineered phages in precision medicine [61]. Beyond clinical applications, recent environmental studies have uncovered previously unknown bacteriophages in everyday items such as toothbrushes, expanding our understanding of phage biodiversity and their biotechnological applications [64]. Additionally, researchers have developed phage-based nanocarriers capable of delivering penicillin into beta-lactam-resistant Gram-negative bacteria, reviving the use of traditional antibiotics through targeted delivery systems [65]. In parallel, consumer-oriented innovations such as a phage topical gel for acne, targeting *Cutibacterium acnes*, are demonstrating rapid and effective results, highlighting the growing commercial and therapeutic versatility of phage technologies [66]. Despite these significant breakthroughs, Bulgaria’s bacteriophage research remains largely rooted in historical studies, lacking the modern genomic, immunological, and clinical methodologies that are now standard in global bacteriophage research. Unlike countries currently integrating phage therapy into clinical trials, personalized medicine, and synthetic biology, Bulgaria has yet to translate its foundational research into tangible medical applications. As nations worldwide push bacteriophage therapy toward regulatory approval and widespread implementation, Bulgaria must reassess its past research and embrace modern phage technologies to remain a relevant player in the fight against antibiotic-resistant infections.

#### 1.3.3. Bacteriophage Therapy and Regulatory Oversight in the EU

The European Union has developed a structured and evolving regulatory framework for bacteriophage therapy, recognizing its potential in combating antibiotic resistance [67]. Phage therapies for human use are regulated under the same legislation as conventional medicines, while veterinary applications follow specific EMA guidelines [68].

A key advancement is the introduction of Chapter 5.31 in the European Pharmacopoeia, which outlines quality standards for Phage Therapy Medicinal Products (PTMPs) [69]. These include propagation in well-characterized bacterial hosts, purification through validated techniques, and adherence to Good Manufacturing Practices. Bacterial cell banks and phage seed lots must be clonally derived and free from harmful genetic elements such as toxins or resistance genes, and are rigorously tested for identity, purity, and stability (EDQM, 2024) [69]. The EU also applies a risk-based quality assurance approach, adjusting the level of control based on patient safety risks, including contamination and endotoxins. Personalized treatments are supported through regulatory pathways like Belgium’s magistral preparations, allowing patient-specific formulations without full market authorization (Comparison between American and European Legislation, n.d.) [69].

Compared to the U.S., the EU approach stands out for its pharmacopeial detail and adaptable risk-based oversight, contributing to safer and more consistent phage therapy products (Comparison between American and European Legislation, n.d.; EDQM, 2024) [69].

Alongside the EU’s evolving regulatory landscape, efforts are also underway to standardize how bacteriophage efficacy is assessed in the laboratory. The European Committee on Antimicrobial Susceptibility Testing (EUCAST) has formed a dedicated subcommittee focused on the development of reliable and harmonized methods for phage susceptibility testing [70]. This initiative aims to establish consistent laboratory practices that can support both research and clinical use of phages, particularly in the treatment of antibiotic-resistant infections. By defining testing protocols, interpretative criteria, and quality control measures, EUCAST seeks to enhance comparability of results across institutions and ensure scientific rigor. These efforts are especially important given the personalized nature of phage therapy, where selecting the right phage for a specific bacterial strain is essential. The standardization of susceptibility testing not only facilitates clinical decision-making but also supports future regulatory approvals and integration of phage-based products into routine medical practice. In this way, EUCAST’s work complements the pharmacopeial and legislative advances already shaping the EU’s approach to phage therapy.

#### 1.3.4. Revisiting Bulgarian Phage Research

All these studies, despite their age, provide valuable insights into Bulgarian bacteriophage research. By integrating past and present, this review enriches the understanding of bacteriophage therapy as a powerful tool against antibiotic-resistant bacteria and phage-host interactions. It is vital to continue evaluating the efficacy and safety of both historical and commercially available bacteriophages, particularly in a concentration-dependent manner. While phages have shown great promise, ensuring their safety at varying concentrations is essential for clinical integration—especially in applications such as wound treatment, combating antibiotic-resistant bacteria, and combining phages with mesenchymal stem cells (MSCs) in regenerative medicine [16,71]. Advances in synthetic biology and combination therapies highlight substantial progress [72,73], but the complex nature of clinical environments, individual variability in patient responses, and potential long-term effects demand rigorous testing to eliminate risks and maximize therapeutic benefits [5,74]. Comprehensive preclinical and clinical evaluations remain vital to validate safety profiles and optimize the integration of phage-based interventions into mainstream medicine.

Given Bulgaria’s historical contributions to phage science, further studies are needed to establish therapeutic safety and efficacy. While countries like Georgia, Poland, and Russia have long incorporated phage therapy into medical practice [75,76], and nations such as Belgium, France, Sweden, Australia, and the United Kingdom have launched national phage therapy initiatives [77], Bulgaria has yet to adopt this approach. This hesitation is due in part to unresolved concerns about cytotoxicity and therapeutic viability. Although existing evidence suggests that bacteriophages themselves generally do not exhibit cytotoxic or cytostatic effects on mammalian cell lines [60,78,79], the potential impact of phage formulations containing stabilizers and excipients remains underexplored [80]. A critical next step would be to evaluate the interactions between bacteriophage-based medications and human cell models to ensure their clinical safety and effectiveness. Conducting controlled cytotoxicity assessments on relevant human cell lines could provide the necessary validation for the medical use of phages in Bulgaria—bridging the gap between theoretical potential and clinical implementation.

Demonstrating the non-cytotoxic nature and further validating the effectiveness of bacteriophages in Bulgaria is essential, as their therapeutic potential spans multiple medical fields and aligns with the country’s evolving treatment strategies. Phage therapy is gaining traction as a promising alternative, particularly for addressing antibiotic-resistant infections and biofilm-related diseases, areas where Bulgaria stands to benefit significantly. For instance, Boyanova in 2023 highlighted the potential of bacteriophages as an innovative treatment for *C. acnes* in acne, offering an alternative to standard dermatological therapies [81]. Likewise, a recent study published in 2024 by Kizheva et al. at the Sofia University “St. Kliment Ohridski” investigated the possible application strategies of phages towards overcoming bacterial resistance in regard to plant protection as substitutes for chemical pesticides, as well as in food safety, in aquatic systems, and animal and human healthcare [82]. However, in order to fully integrate bacteriophage-based treatments into contemporary Bulgarian medicine, comprehensive cytotoxicity and efficacy evaluations are crucial.

#### 1.3.5. Today’s Application and Challenges in Phage Therapy

Bacteriophage therapy holds significant promise as a targeted and effective alternative to traditional antibiotics, particularly in the fight against multidrug-resistant bacterial infections. Phages, naturally present in the environment and human microbiota, are generally safe, compared to antibiotic chemotherapy, with minimal side effects such as allergic reactions [20]. Their high specificity allows for precise targeting of bacterial pathogens, making them useful not only in therapy but also in applications such as bacterial typing, genetic engineering, and vaccine development [1,76]. Some studies even suggest their potential in cancer therapy and as models for latent infection studies [83,84,85]. However, despite these advantages, several challenges continue to limit the widespread clinical adoption of phage therapy. One major issue is the absence of globally harmonized regulatory frameworks, which complicates the standardization of phage production, quality control, and clinical evaluation [86]. The strict host specificity of phages, while a benefit in targeting, is also a limitation in practice, as it requires accurate and timely identification of the causative pathogen. In many clinical settings, this precision is not always feasible, especially in acute infections [4]. To overcome this, phage cocktails are developed, but their formulation introduces additional complexity, as inter-phage interactions must be considered and validated [6].

Other concerns include the potential for phages to transfer virulence factors or antibiotic resistance genes through horizontal gene transfer, although this remains a topic of ongoing debate [87]. Additionally, the natural decline of phage concentrations in the human body and variable immune responses can impact treatment effectiveness [88]. Moreover, there is currently a lack of standardized methods for large-scale phage production that ensure safety, genetic stability, and cost-effectiveness [89,90]. Public perception also plays a role, as phages are often misunderstood as “dangerous viruses,” and greater awareness among healthcare providers and patients is essential [91].

Future research in the field of bacteriophage, particularly in Bulgaria, holds significant potential despite existing challenges. The country’s strong academic tradition, emerging biotech industry, and strategic European position offer a promising foundation for innovation. With focused investment, international collaboration, and supportive regulatory policies, Bulgaria could play a pivotal role in advancing phage therapy research and its clinical translation.

## 2. Conclusions

Bacteriophage therapy offers a compelling solution to the pressing issue of antibiotic resistance by providing targeted bacterial control while preserving beneficial microbiota. The insights from this review emphasize the importance of leveraging lytic phages’ therapeutic potential and ensuring the safety of commercial preparations through rigorous evaluation. Bulgaria’s contributions, both historical and recent, reflect the nation’s untapped capacity for advancing bacteriophage research and innovation. By bridging historical research with contemporary molecular approaches, Bulgaria is well-positioned to revive its phage research legacy and make meaningful contributions to the global fight against antimicrobial resistance. However, for phage therapy to realize its full potential, challenges such as standardizing production protocols, addressing regulatory gaps, and managing bacterial resistance must be overcome. By fostering international collaborations, investing in infrastructure, and establishing supportive policies, bacteriophage therapy can emerge as a critical tool for managing bacterial infections and reducing the global burden of antibiotic resistance in the future.

## Figures and Tables

**Table 2:** Comparison of the original Aldová bacteriophage enrichment method and the modified protocol used in the 1973 study by Kruchmarova-Raycheva.

Step	Aldová’s Original Method (1964) [44]	Modifications by Kruchmarova—Raycheva (1973) [12]
Sample source	Environmental sources (e.g., sewage, soil)	Clinical isolates from hospitalized patients
Bacterial host	Standard lab strains	Clinical strains on *K. pneumoniae* and *E. aerogenes*
Incubation conditions	Unspecified standard times and temperatures	Unchanged (sterile filtration to isolate phages)
Filtration	Standard bacterial filtration	Followed by detailed host range testing
Detection	Plaque formation on bacterial lawn	Included phage typing using clinical stain panels

## Data Availability

No new data were created or analyzed in this study.
